# Tailoring the Mechanical Properties of Laser Cladding-Deposited Ferrous Alloys with a Mixture of 410L Alloy and Fe–Cr–B–Si–Mo Alloy Powders

**DOI:** 10.3390/ma12030410

**Published:** 2019-01-29

**Authors:** Sheng Huang, Dichen Li, Lianzhong Zhang, Xiaoyu Zhang, Weijun Zhu

**Affiliations:** 1 State Key Laboratory for Manufacturing Systems Engineering, Xi’an Jiaotong University, Xi’an 710049, China; ahhuangsheng@126.com (S.H.); zhanglianzhong11@sina.com (L.Z.); zhangxiaoyu94@163.com (X.Z.); zhuweijun99@sina.com (W.Z.); 2 School of Mechanical Engineering, Xi’an Jiaotong University, Xi’an 710049, China

**Keywords:** additive manufacturing, laser cladding deposition, 410L alloy, Fe–Cr–B–Si–Mo alloy, microstructure, mechanical properties

## Abstract

The effects of different ratios of 410L alloy and Fe–Cr–B–Si–Mo alloy powders on the microstructure and mechanical properties of laser cladding-deposited ferrous alloys were investigated. The experimental results revealed that the 410L alloy had good strength and excellent ductility due to its microstructure consisting of large elongated ferrite dendrites surrounded by a small number of martensite grains, while the Fe–Cr–B–Si–Mo alloy had high strength and poor ductility because of its eutectic microstructure composed of ferrite and Fe_2_B/Cr_2_B. As the concentration of Fe–Cr–B–Si–Mo alloy powder added to the 410L alloy powder increased, the ferrite grains became finer and the volume fraction of the eutectic increased, which eventually improved the strength and reduced the plasticity. Then, 410L + 12.5% Fe–Cr–B–Si–Mo alloy powder was successfully deposited onto AISI 1060 steel substrate via laser cladding deposition, and the mechanical properties met those of the substrate, which verified that tailoring the mechanical properties of the laser cladding-deposited alloys with a mixture of 410L and Fe–Cr–B–Si–Mo alloy powders for steel repairing applications is a feasible solution.

## 1. Introduction

Many defective components, which are mis-machined in the manufacturing process or locally worn in the service process, have to be scrapped, resulting in serious resource waste and huge economic losses. Steel is the most commonly used materials for gears, shafts, railway wheels, and so on in many industrial applications, and the market demand for the repairing of such products is huge. Therefore, it is urgently necessary to find an effective repair method to restore the geometry and provide sufficient strength to such components, allowing for their continued use [1].

Laser cladding deposition (LCD), also called laser engineering net shaping (LENS) [2], is a laser additive manufacturing process, which is based on the iterative process of coaxial powder delivery melting/rapid solidification layer-by layer deposition [3]. The deposited layers are metallurgically bonded and exhibit a narrow heat-affected zone and small dilution zones, possessing a high accuracy and outstanding mechanical property [4]. Owing to the manufacturing principle and those significant merits, LCD technique is suitable for the repairing of defective components [5]. 

Repairing steel parts using LCD has been the subject of much research in recent years. Liu et al. [4,6] presented a feasibility exploration of superalloys for AISI 4140 steel repairing using LENS. However, the tensile strength and hardness value of the specimens did not meet the industrial requirements. Liu et al. [5] applied the Taguchi method to optimize the process parameters of Fe-based self-fluxing alloy for the repair of an AISI 4140 sprocket using laser cladding. The repair accuracy of the sprocket could reach 2.973 mm, and metallurgical bonding was realized between the cladding zone and the base metal. Marya et al. [7] demonstrated that the direct energy deposition repair of ferrous parts with UNS N06625 might restore damaged surfaces. Sun et al. [8] utilized laser cladding technology to repair 300M steel with 420 stainless steel powder and incorporated a laser idle time between each clad track to control the in situ quench and tempering sequence. It was found that the ductility was significantly improved. Xu et al. [9] applied simulation and experiments to study the remanufacturing process of a disabled thin-wall FV520B steel impeller blade with Fe–Cr–Ni–Cu alloy powder using laser cladding, and defects like pores, cracks, or slag inclusions were not detected in the remanufactured region. Lewis et al. [10] assessed the wear and rolling contact fatigue performance of laser cladded-rail with four materials. The results showed that the rail cladded by 316 stainless steels did not deform under the cyclic loading applied and would offer a greatly enhanced rolling contact fatigue life. Lai et al. [11,12] investigated the effects of the cladding direction, preheating, and post-heat treatment on the microstructure and mechanical properties of laser cladding-repaired rail with 410L alloy. An excellent microstructural consistency was established across the railhead and its heat-affected zone.

Although many studies have been published concerning the restoration of the geometry and surface properties of steel parts, such as wear and erosion properties for laser cladding repair, [13], little attention has been paid to the mechanical properties of repaired steel components. The method of matching of the mechanical properties between the repair material and the substrate is important for the performance of the parts after repair. Meanwhile, the ability to join dissimilar metals represents a significant challenge due to the residual stresses [14], delamination [15], and intermetallic compounds, which might appear in LCD and render the material brittle [16]. 410L and Fe–Cr–B–Si–Mo alloys are both ferritic stainless steel and suitable cladding materials for the repairing of steel parts owing to their great metallurgical compatibility with steels [11,12] and excellent laser compatibility [15,17,18,19,20]. 410L alloy is widely used in automobile exhaust gas treatment devices and boiler combustion chambers because of its outstanding bending performance, ductility, high-temperature oxidation resistance. Fe–Cr–B–Si–Mo alloy is used for wear-resistant coatings of shafts due to its high toughness and crack resistance. However, the mechanical properties of the two alloys do not match those of most steels, such as AISI 5140 steel, AISI 1060 steel, and so on.

A large number of damaged steel parts have to be repaired every year. However, there are many kinds of steel. Each steel part needs to be repaired with one material, whose mechanical properties are similar to the mechanical properties of the steel part, limiting the development of the repair industry. The purpose of this paper is to present a new method for repairing varied kinds of steel with only two materials. The proposed method is to tailor the mechanical properties of ferrous alloys with a mixture of 410L and Fe–Cr–B–Si–Mo alloy powders via laser cladding deposition, such that the mechanical property meets the requirements of the steel parts. The microstructure and mechanical properties of the laser-deposited 410L alloy with different concentrations of Fe–Cr–B–Si–Mo powder were investigated. Then, a case study of AISI 1060 steel repairing was implemented with the suitable powder, whose mechanical properties are similar to those of the substrate.

## 2. Materials and Methods 

**Figure 1:**
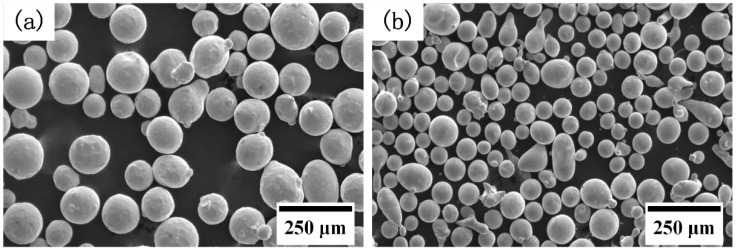
SEM micrographs of (**a**) 410L powder and (**b**) Fe–Cr–B–Si–Mo powder.

Gas-atomized 410L powder (Höganäs AB, Höganäs, Sweden) with a particle size range of 53–150 μm and gas-atomized Fe–Cr–B–Si–Mo powder (BGRIMM Advanced Materials Science & Technology Co,. Ltd, Beijing, China) with a particle size range of 45–105 μm were employed in the present work. The morphologies of the 410L and Fe–Cr–B–Si–Mo powders are shown in Figure 1. The substrate used was AISI 5140 steel (Shandong Iron & Steel Group Co., Ltd, Jinan, China), which was 150 mm × 100 mm × 10 mm in size. The chemical compositions are given in Table 1. To investigate the effect of the concentration of Fe–Cr–B–Si–Mo powder on the microstructure and mechanical properties of the alloys, 5 kinds of powders, 410L, 410L + 6.25% Fe–Cr–B–Si–Mo, 410L + 12.5% Fe–Cr–B–Si–Mo, 410L + 25% Fe–Cr–B–Si–Mo, and Fe–Cr–B–Si–Mo, respectively named A1, A2, A3, A4, and A5, were prepared for LCD experiments.

**Figure 2:**
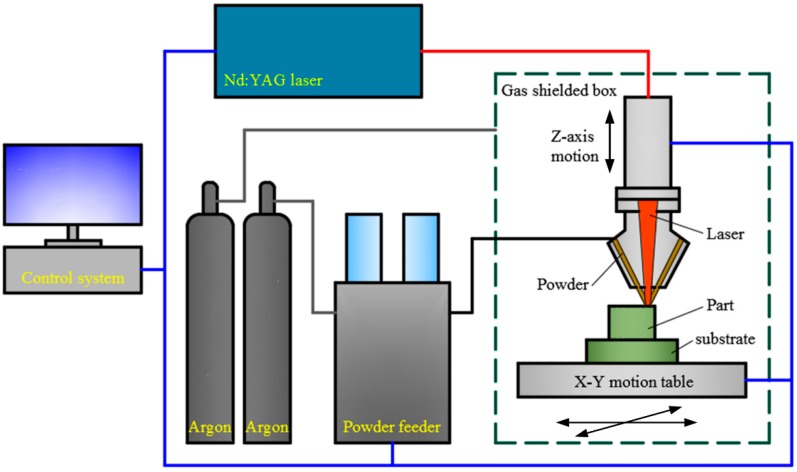
Schematic diagram of the laser cladding deposition (LCD) system.

All the alloy samples were deposited using a homemade LCD system (Xi’an Jiaotong University, Xi’an, China) [21]. The schematic diagram of the LCD system is illustrated in Figure 2. The system fundamentally consists of a 1000 W Nd:YAG laser, a coaxial powder feed system, a gas shielded box, and a 3-axis motion control system. Argon was used as the powder carrier gas and shielding gas during the LCD process. The powder was uniformly mixed and dried, and the substrate surface was polished and cleaned by ethanol before the deposition. The optimized process parameters were employed, as shown in Table 2. The hatch path was set to an S shape, and the scanning direction of each layer was perpendicular to the previous layer. A rectangle sample of 15 mm × 9 mm × 5 mm, and 3 cylindrical samples of Φ 8.2 mm × 45 mm for each alloy were built via LCD.

The rectangle samples were sectioned vertically through the middle in length. The sections parallel and perpendicular to the building direction were mounted, grinded, and polished for metallographic observations and phase composition analysis, respectively. The metallographic specimens were then etched with a solution of 5 g CuCl_2_, 100 mL HCl, and 100 mL ethanol. The cylindrical samples were machined into dogbone-shaped tensile specimens with a gauge section of 20 mm (length) × 3 mm (diameter). The tensile tests were performed by an Instron 5985 tensile machine (Instron Corporation, Boston, America) at room temperature (22 °C). The etched metallographic specimens and the fracture surfaces of the tensile specimens were evaluated by a Hitachi SU-8010 scanning electron microscope (SEM, Hitachi, Ltd., Tokyo, Japan). The phase compositions of the sections, which were perpendicular to the building direction and were not etched, were identified by X-ray diffraction (XRD, Bruker AXS GmbH, Karlsruhe, Germany), using a Bruker D8 Diffractometer (Bruker AXS GmbH, Karlsruhe, Germany) with Cu Kα radiation.

## 3. Results and Discussion

### 3.1. Microstructural Evaluation

**Figure 3:**
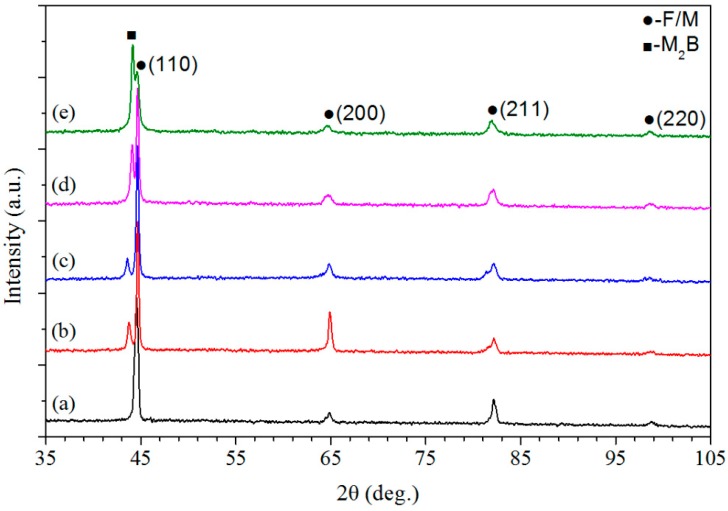
X-ray diffraction patterns of deposited alloys with different weight fractions of Fe–Cr–B–Si–Mo powder: (**a**) 0%, (**b**) 6.25%, (**c**) 12.5%, (**d**) 25%, and (**e**) 100%, respectively.

Figure 3 shows the X-ray diffraction patterns of the deposited alloys with different weight fractions of Fe–Cr–B–Si–Mo powder. From Figure 3, it can be noted that the peaks of ferrite/martensite (F/M) were all recorded in the pattern of the deposited alloys. Only the F/M peaks were observed in the pattern of the 410L alloy, as shown in Figure 3a, and the result is in agreement with the literature [11,12]. Figure 3b–d show the X-ray diffraction patterns of the deposited alloys produced with a mixture of 410L and Fe–Cr–B–Si–Mo alloy powders. Compared with the patterns of 410L alloy, in addition to the peaks of F/M, the peaks of Fe_2_B/Cr_2_B (M_2_B) were also recorded, the peak intensities of M_2_B increased with the weight fraction of Fe–Cr–B–Si–Mo in 410L, which corresponded to the volume fraction of the M_2_B phase increase. Figure 3e shows the X-ray diffraction patterns of the Fe–Cr–B–Si–Mo alloy. From Figure 3e, it can be observed that the peak intensities of M_2_B were prominently higher than those of F/M, which suggests that the volume fraction of the M_2_B phase may be more than that of the F/M phase.

**Figure 4:**
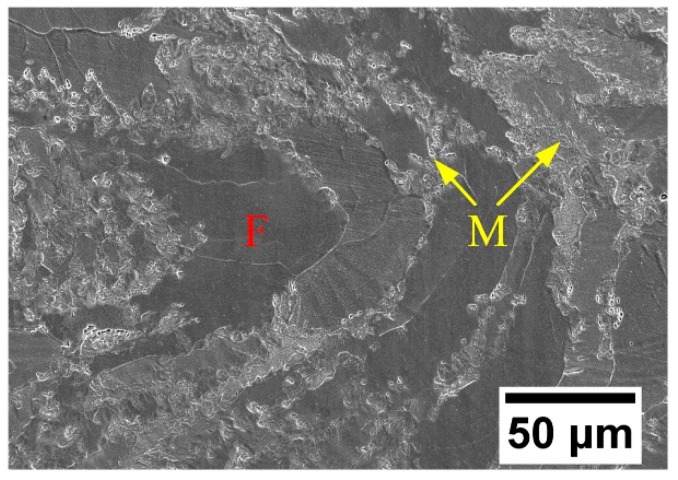
SEM micrograph of the deposited 410L alloy.

Figure 4 shows the microstructural characteristics of the 410L alloy. From Figure 4, it can be seen that there were large, elongated ferrite grains (dark color) and a small quantity of martensite grains (bright color), which were distributed along the ferrite grain boundary. This is due to the fact that during the transformation from austenite to ferrite, the ferrite nuclei form at the austenitic grain boundary and coarsen into the interior; meanwhile, the carbon diffuses into the austenite, which increases the austenitic carbon content and promotes the transformation of austenite to martensite. Although silicon was contained in the 410L alloy, no eutectic microstructure was observed.

**Figure 5:**
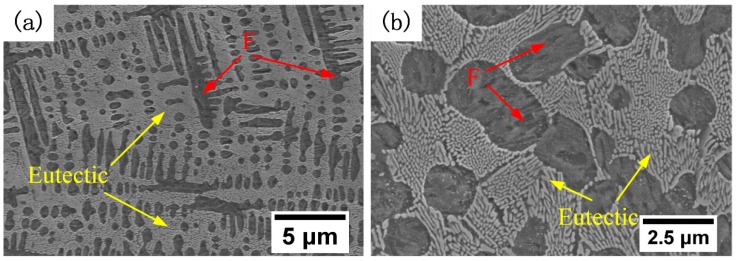
SEM micrographs of the deposited Fe–Cr–B–Si–Mo alloys. (**a**) Low- magnification; (**b**) high- magnification.

A hypoeutectic microstructure was detected in the Fe–Cr–B–Si–Mo alloy, as shown in Figure 5. Fine ferrite grains occupy subordinately, while a eutectic microstructure consisting of ferrite and M_2_B (bright color) dominated the alloy, and the volume fraction of the eutectic was greater than 50%. This might be mainly attributed to the boron contained in the Fe–Cr–B–Si–Mo alloy; the boron exists in liquid near the liquid–solid interfaces during the solidification process due to its weak solubility in solid iron, which increases the constitutional undercooling of the remaining liquid, promotes the eutectic reaction [22], and restrains the growth of austenite grains. Meanwhile molybdenum contained in the Fe–Cr–B–Si–Mo alloy also acts to refine the austenite grains. Fine austenite grains eventually transform into fine ferrite grains during cooling.

**Figure 6:**
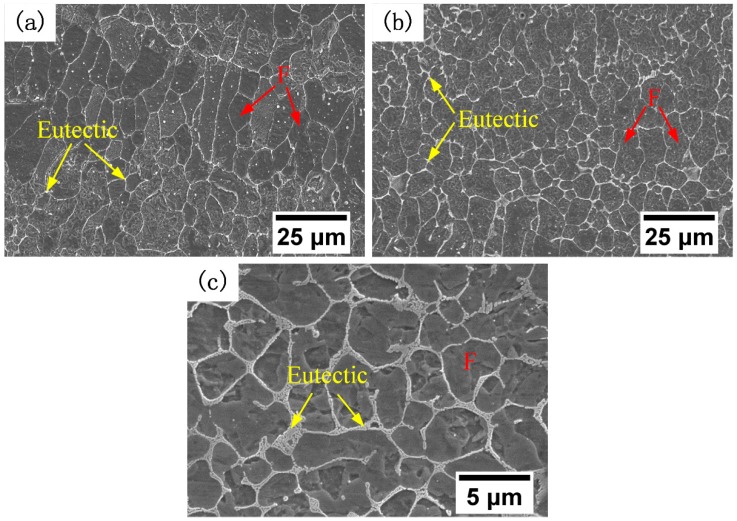
SEM micrographs of the deposited alloys at different concentrations of Fe–Cr–B–Si–Mo powder: (**a**) 6.25%, (**b**) 12.5%, and (**c**) 25%, respectively.

**Figure 7:**
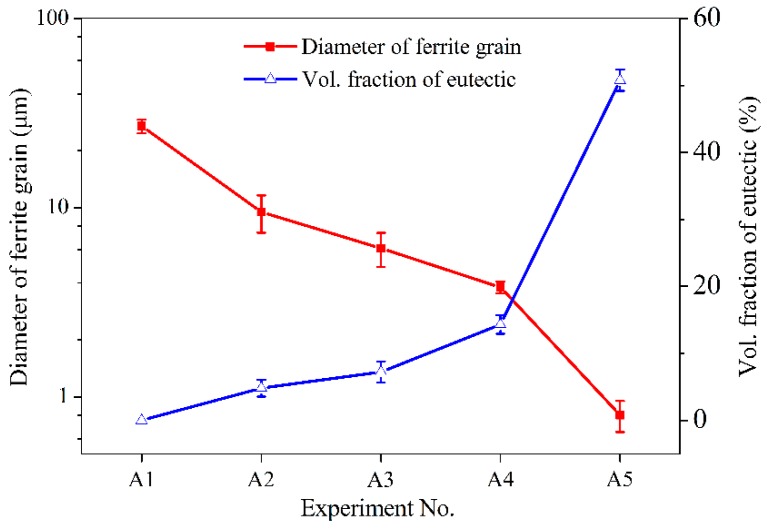
Diameter of ferrite grain and volume fraction of the eutectic in the deposited alloys at different concentrations of Fe–Cr–B–Si–Mo powder.

Micrographs of a typical cross section of the deposited alloys with different concentrations of Fe–Cr–B–Si–Mo powder are shown in Figure 6. The diameter of the ferrite grains and the volume fraction of the eutectic in Figure 4, Figure 5 and Figure 6 were calculated using ImageJ software, as shown in Figure 7. Fine ferrite grains and a small amount of interdendritic divorced eutectic were observed in the deposited 410L + 6.25% Fe–Cr–B–Si–Mo, as shown in Figure 6a. The size of the ferrite grains reduced to one-third of that of the 410L alloy (Figure 4). The ferrite grain size decreased, while the volume fraction of the eutectic increased with the increasing weight fraction of Fe–Cr–B–Si–Mo in the 410L alloy, as shown in Figure 7, and similar results were also observed in the literature [22]. This may be due to the fact that the liquid phase composition is closer to the eutectic composition as the boron content increases, so the volume fraction of the eutectic is larger, and the size of ferrite grains transformed from austenite is finer.

**Figure 8:**
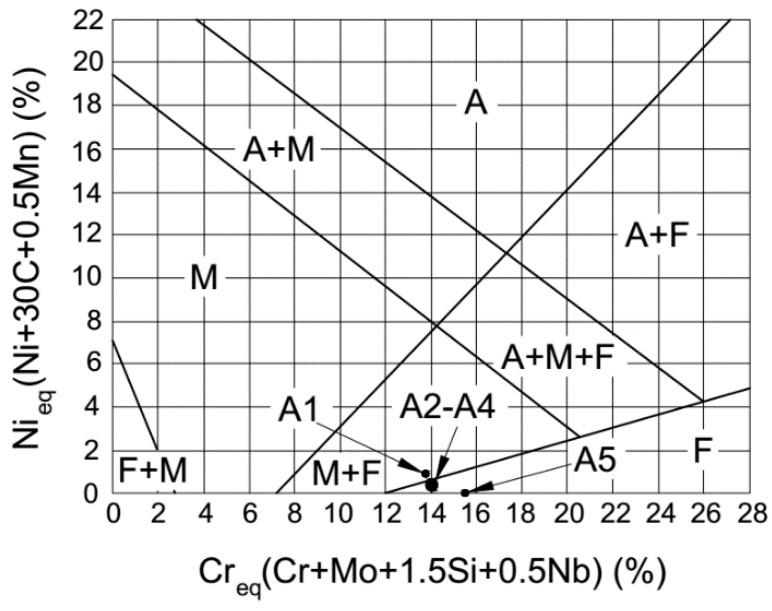
Ferrite and martensite distribution of the deposited samples on the Schaeffler diagram.

Further, no martensite phase was observed in the deposited alloys containing Fe–Cr–B–Si–Mo alloy, as shown in Figure 5 and Figure 6. To illustrate this phenomenon, the Schaeffler diagram was adopted, as shown in Figure 8. The chemical composition of ferritic stainless-steel elements (except Fe) are simplified by the chromium equivalent (*Cr_eq_*) and nickel equivalent (*Ni_eq_*), and the *Cr_eq_* and *Ni_eq_* are shown below:

(1) $$Cr_{eq}=Cr+Mo+1.5Si+0.5Nb$$

(2) $$Ni_{eq}=Ni+30C+0.5Mn$$

where each chemical element symbol indicates the percentage content [23]. Ferrite and martensite distribution in each alloy can be found in the Schaeffler diagram, except for the 410L alloy (A1); the other alloys (A2–A5) are located in the ferrite region of the Schaeffler diagram due to the content of carbon and manganese decreasing when the Fe–Cr–B–Si–Mo alloy was mixed into the 410L alloy.

### 3.2. Tensile Properties

**Figure 9:**
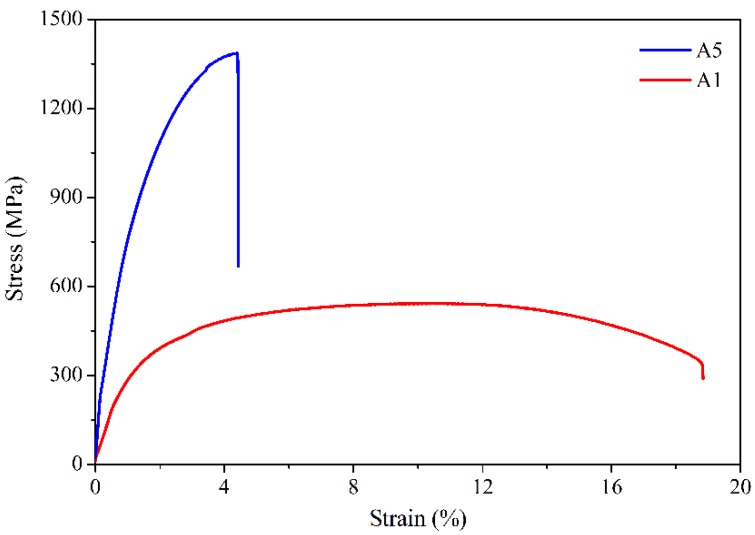
Stress–strain curves of the deposited 410L alloy and Fe–Cr–B–Si–Mo alloy specimens.

**Figure 10:**
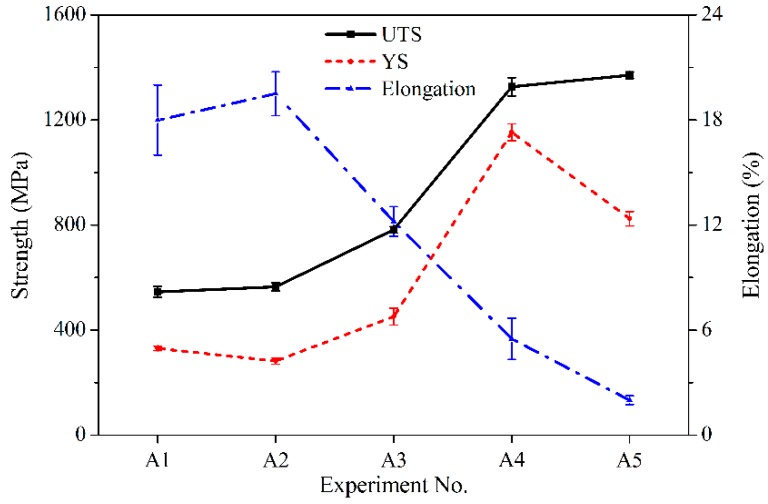
Ultimate tensile strength, yield strength, and elongation of deposited alloys at different concentrations of Fe–Cr–B–Si–Mo powder.

Figure 9 shows the stress–strain curves of the 410L alloy and Fe–Cr–B–Si–Mo alloy. From Figure 9, it can be observed that the yield strength (YS), ultimate tensile strength (UTS), and elongation of 410L alloy were 330 MPa, 545.5 MPa, and 18%, respectively, and the excellent tensile properties were attributed to its microstructure composed of ferrite and martensite. The YS, UTS, and elongation of the Fe–Cr–B–Si–Mo alloy were 841.5 MPa, 1365.5 MPa, and 2%, respectively, and the high strength and low plasticity were due to the eutectic microstructure. The tensile results of the deposited alloys with different weight fractions of Fe–Cr–B–Si–Mo powder are shown in Figure 10. In Figure 10, as the mass fraction of Fe–Cr–B–Si–Mo mixed in the 410L alloy increases, the YS and UTS increases from 282 MPa to 1153.3 MPa and from 565 MPa to 1326.7 MPa, respectively, and the elongation decreases from 22% to 5.5%. This is related to the aforementioned changes in the microstructure of the alloys; the strength of the alloy is improved because of the ferrite grain refinement according to the Hall–Petch strengthening behavior and the increasing volume fraction of the eutectic, while the plasticity is reduced due to the increasing numbers of the eutectic.

**Figure 11:**
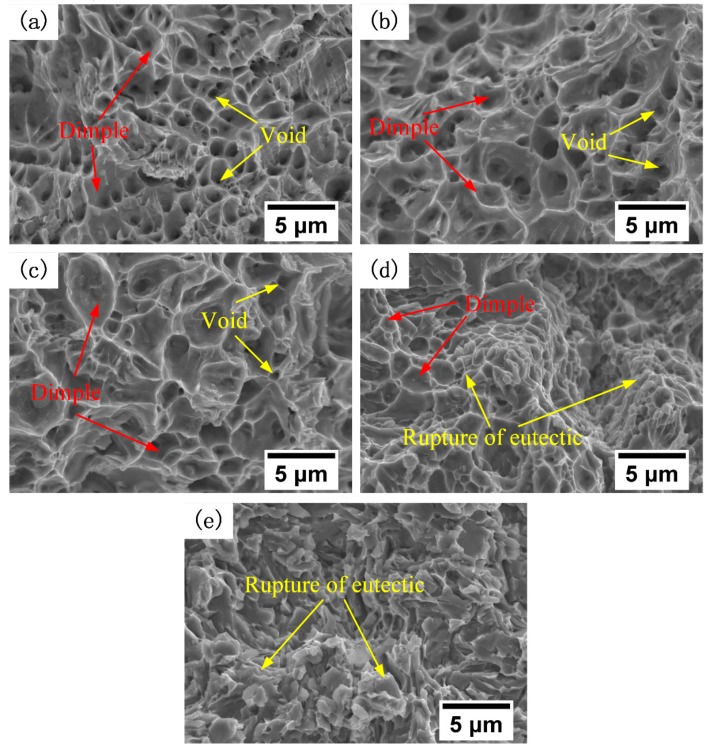
SEM micrographs of the fracture surface of deposited (**a**) 410L, (**b**) 410L + 6.25% Fe–Cr–B–Si–Mo, (**c**) 410L + 12.5% Fe–Cr–B–Si–Mo, (**d**) 410L + 25% Fe–Cr–B–Si–Mo, and (**e**) Fe–Cr–B–Si–Mo alloys, respectively.

The fracture surfaces were examined using SEM to investigate the different tensile properties of the alloys (Figure 11). The SEM analysis showed that the tensile fracture of the alloys changed from ductility fracture to cleavage fracture. Many fine dimples and microvoids were observed on the fracture surfaces of the 410L alloy (Figure 11a) and the 410L + 6.25% Fe–Cr–B–Si–Mo alloy (Figure 11b), which indicated that the fracture surfaces were ductile, the ductile fractures were reasonably consistent with the high elongation of the 410L alloy and the 410L + 6.25% Fe–Cr–B–Si–Mo alloy (Figure 10). Figure 11c presents the fracture surface of the 410L + 12.5% Fe–Cr–B–Si–Mo alloy; it can be seen that fewer and shallower dimples existed on the fracture surface of the 410L + 12.5% Fe–Cr–B–Si–Mo alloy compared with that of the 410L alloy and the 410L + 6.25% Fe–Cr–B–Si–Mo alloy. The fracture of the 410L + 25% Fe–Cr–B–Si–Mo alloy was a mixture of quasi-cleavage fracture and ductile fracture (Figure 11d). The fracture of the Fe–Cr–B–Si–Mo alloy was observed to be in quasi-cleavage failure mode (Figure 11e).

### 3.3. A Case Study of the Repair of AISI 1060 Steel

**Figure 12:**
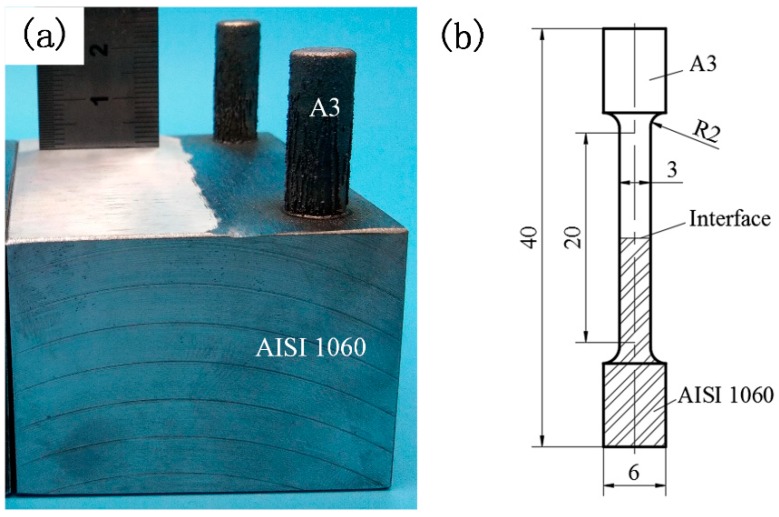
(**a**) Representative specimens after the 410L + 12.5% Fe–Cr–B–Si–Mo was deposited onto the AISI 1060 steel substrate; (**b**) dimensions of the tensile samples of repair.

**Figure 13:**
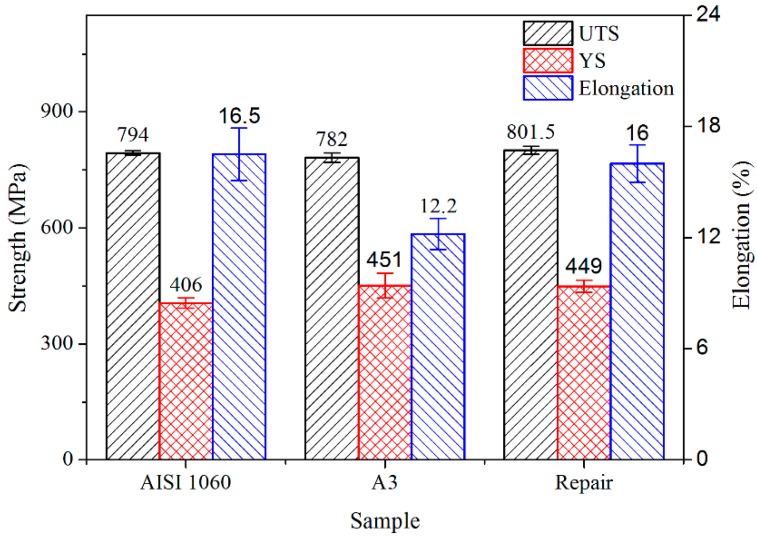
Comparison of the mechanical properties of the AISI 1060, 410L + 12.5% Fe–Cr–B–Si–Mo, and repair samples.

The above research results showed that the mechanical properties of the deposited alloys could be adjusted by adding Fe–Cr–B–Si–Mo powder to the 410L alloy, which matched the mechanical properties of the steel that needed to be repaired. A case study of the repair of AISI 1060 steel (Table 1) was used to verify the feasibility of this method. AISI 1060 steel is a material used for railway wheels, which is usually worn and deformed by the complex contact between the wheels and rails, and the worn wheel is difficult to repair. The mechanical properties of AISI 1060 steel were close to those of the deposited 410L + 12.5% Fe–Cr–B–Si–Mo, so the 410L + 12.5% Fe–Cr–B–Si–Mo powder was chosen as the repair material, and three 410L + 12.5% Fe–Cr–B–Si–Mo pillars (length of 25 mm and diameter of 8.2 mm) were deposited onto the AISI 1060 steel substrate (dimensions of 75 mm × 55 mm × 40 mm) (Figure 12a). Then, the specimen was machined onto the tensile samples. The size of the tensile samples is shown in Figure 12b. The mechanical properties of the AISI 1060 steel substrate samples, deposited 410L + 12.5% Fe–Cr–B–Si–Mo samples, and 410L + 12.5% Fe–Cr–B–Si–Mo for the AISI 1060 steel repairing samples are shown in Figure 13. From Figure 13, it can be noted that a YS of 406 MPa, an UTS of 794 MPa, and an elongation of 16.5% were obtained for the AISI 1060 steel substrate. Compared with the AISI 1060 steel substrate, the YS of the 410L + 12.5% Fe–Cr–B–Si–Mo for AISI 1060 steel repairing was 449 MPa, about 110% of the YS of the substrate, the UTS was 801.5 MPa, slightly higher than that of the substrate, and the elongation was 16%, about 97% of the elongation of the substrate, which indicated that the mechanical properties might meet the requirements for AISI 1060 steel repairing.

## 4. Conclusions

In this study, the microstructure features and mechanical properties of cladding with a mixture of 410L alloy and Fe–Cr–B–Si–Mo alloy powders were studied, and then, a case study of 410L + 12.5% Fe–Cr–B–Si–Mo repairing of AISI 1060 steel was implemented. The conclusions are as follows:

1. Coatings with several combinations of strength and plasticity were manufactured using LCD by adding different proportions of Fe–Cr–B–Si–Mo powder to 410L powder, which are available for the repair of various kinds of steel parts.
2. The microstructural morphologies of the deposited 410L alloy chiefly consisted of large, elongated ferrite grains and a small number of martensite grains distributed around the ferrite grain boundary, and it has low strength and good ductility.
3. There were fine equiaxed ferrite grains and eutectic microstructures composed of ferrite and Fe_2_B/Cr_2_B in the deposited Fe–Cr–B–Si–Mo alloy, and no martensite phases were observed. The alloy has high strength and low ductility.
4. With the increasing mass fraction of Fe–Cr–B–Si–Mo in the 410L alloy, the ferrite grains became finer, and the volume fraction of the eutectic increased. Moreover, the yield strength and ultimate tensile strength increased, and the elongation decreased.
5. AISI 1060 steel was successfully repaired by 410L + 12.5% Fe–Cr–B–Si–Mo via LCD, and the mechanical properties of the substrate + deposited alloy were similar to those of the AISI 1060 steel substrate. This shows that tailoring the mechanical properties of laser cladding-deposited alloys with a mixture of 410L alloy and Fe–Cr–B–Si–Mo alloy powders for steel repairing is a feasible solution.

## Figures and Tables

**Table 1:** Chemical composition of 410L, Fe–Cr–B–Si–Mo , and AISI 5140 and AISI 1060 steel alloys (wt %).

Materials	Cr	B	Si	C	Mn	Mo	Ni	Cu	Fe
410L	13	-	0.5	0.03	0.1	-	-	-	Balance
Fe–Cr–B–Si–Mo	13	1.6	1.2	-	-	0.8	-	-	Balance
AISI 5140	0.80	-	0.25	0.40	0.80	-	-	-	Balance
AISI 1060	0.25	-	0.27	0.61	0.65	0.1	0.25	0.25	Balance

**Table 2:** Process parameters of laser cladding deposition.

Process Parameter	Unit	Values
Laser power	W	180
Scanning speed	mm/s	10
Powder flow rate	g/min	4.5
Spot diameter	mm	0.5
Hatch spacing	mm	0.3
Layer thickness	mm	0.1
